# Super-strong magnetic field-dominated ion beam dynamics in focusing plasma devices

**DOI:** 10.1038/s41598-022-10829-1

**Published:** 2022-04-27

**Authors:** A. Morace, Y. Abe, J. J. Honrubia, N. Iwata, Y. Arikawa, Y. Nakata, T. Johzaki, A. Yogo, Y. Sentoku, K. Mima, T. Ma, D. Mariscal, H. Sakagami, T. Norimatsu, K. Tsubakimoto, J. Kawanaka, S. Tokita, N. Miyanaga, H. Shiraga, Y. Sakawa, M. Nakai, H. Azechi, S. Fujioka, R. Kodama

**Affiliations:** 1 Institute of Laser Engineering, Osaka University, Suita, Japan; 2 ETSI Aeronautica y del Espacio, Universidad Politecnica de Madrid, Madrid, Spain; 3 Hiroshima University, Hiroshima, Japan; 4 Lawrence Livermore National Laboratory, Livermore, USA; 5 National Institute of Fusion Science, Toki, Japan

**Keywords:** Astronomy and planetary science, Physics

## Abstract

High energy density physics is the field of physics dedicated to the study of matter and plasmas in extreme conditions of temperature, densities and pressures. It encompasses multiple disciplines such as material science, planetary science, laboratory and astrophysical plasma science. For the latter, high energy density states can be accompanied by extreme radiation environments and super-strong magnetic fields. The creation of high energy density states in the laboratory consists in concentrating/depositing large amounts of energy in a reduced mass, typically solid material sample or dense plasma, over a time shorter than the typical timescales of heat conduction and hydrodynamic expansion. Laser-generated, high current–density ion beams constitute an important tool for the creation of high energy density states in the laboratory. Focusing plasma devices, such as cone-targets are necessary in order to focus and direct these intense beams towards the heating sample or dense plasma, while protecting the proton generation foil from the harsh environments typical of an integrated high-power laser experiment. A full understanding of the ion beam dynamics in focusing devices is therefore necessary in order to properly design and interpret the numerous experiments in the field. In this work, we report a detailed investigation of large-scale, kilojoule-class laser-generated ion beam dynamics in focusing devices and we demonstrate that high-brilliance ion beams compress magnetic fields to amplitudes exceeding tens of kilo-Tesla, which in turn play a dominant role in the focusing process, resulting either in a worsening or enhancement of focusing capabilities depending on the target geometry.

## Introduction

A significant fraction of the visible Universe is composed by matter in extreme conditions of temperature, density and pressure. When the pressure in a physical system exceeds 1 Mbar, this is defined as a high energy density (HED) state, which corresponds to the pressure required to deform the water molecule or in other words the pressure at which water becomes compressible, corresponding to an energy density exceeding 10^11^ J/m^3^^1^.

In nature we can find numerous examples of HED states, from the cores of gas-giant planets where the extreme pressures modify the fundamental properties of hydrogen and water–ice, leading to highly conductive interiors at the origin of the large magnetic fields characteristic of these planets^2–4^, to the interiors of brown dwarfs^5^, stars and more exotic objects like white dwarfs and neutron stars, where super-strong magnetic fields of amplitudes respectively of 10^6^–10^7^ Gauss and 10^11^ Gauss are often associated to these objects and determine the plasma physics of their surroundings^6,7^. These fields are inferred through UV spectroscopy showing extreme Zeeman splitting in the line emission of the plasma ions in the exotic atmospheres of these objects^8^.

High power lasers made possible to recreate HED conditions in the laboratory and begin a more experimental and less observational/deductive study, allowing for example to compress matter to densities and pressures like those found in the interior of giant planets^9^ and to directly conduct measurements that would otherwise be impossible, offering insights into worlds once accessible only theoretically.

One of the modalities to create HED conditions in matter is through ultra-fast heating of material samples and compressed plasmas using high-brilliance laser-generated ion and proton beams.

Since their discovery over two decades ago^10^, high-intensity laser-generated proton and ion beams have been intensively investigated by the scientific community due to their remarkable properties, that make them ideal ion sources for application to High Energy Density (HED) Physics. The acceleration method treated in this work is called “target normal sheath acceleration” (TNSA), where ions from the surface of a thin foil are accelerated by a charge-separation electric field set up by relativistic electrons generated via intense laser-plasma interaction, that propagate through the target volume and expand into vacuum^11,12^.

TNSA proton beams are accelerated to multi-MeV energies in few picoseconds. They carry a significant fraction of the laser energy (0.5 to 10% depending on the laser system and targets), they are highly directional and characterized by a quasi-laminar flow that guarantees their focusability^13,14^. These properties make them suitable sources for ultra-fast heating of solid samples or dense plasmas to tens or hundreds of eV’s, attaining plasma pressures typical of the interior of giant planets, thus allowing the study of the properties and equations of state of matter in these extreme conditions^15,16^.

Here we focus our attention to the application of intense proton beams to the creation of HED states, and on their control and focusing by plasma devices such as cone targets.

This type of devices was first envisioned for application to relativistic electron-based Fast Ignition research^17^, where the function of the cone is simply to maintain a clear path for the ultra-intense laser from the ablation plasma originating from the implosion of the fusion fuel capsule. They subsequently were re-proposed for ion/proton based Fast Ignition^18,19^, with the addition of a hemispherical shell (hemi) to generate the ion beam, mounted on the cone inner wall at about 300 µm from the cone tip. In this case the cone has two functions: keep the clear path for the intense laser as mentioned before and focus down the proton beam towards the tip of the cone by charge separation electric fields arising from the fast electron propagation along the cone wall.

Several works have been dedicated to the physics of cone targets for ion focusing^20–22^ and in particular a seminal work by Bartal et al.^23^ investigated the proton focusing with tip-less cone targets and determined that electric fields play a dominant role both inside the cone, as focusing field, and outside the cone where the focused proton beam is subject to hot electron pressure that results in a defocusing, radial electric field surrounding the ion beam waist, ultimately driving its expansion.

In this work we show that previous results represent a partial picture of the proton beam dynamics in focusing devices and are limited to relatively low-drive laser energies and rather large-aperture short-length cone targets.

For higher laser energies and cone geometries more appropriate to high-temperature plasma heating and inertial fusion experiments, however, we demonstrate that return current-generated magnetic fields play a dominant role in the proton beam dynamics, constituting the root-cause of proton beam focusing to the cone tip and that, depending on the cone geometry, either worsen, resulting in a ring-like proton emission, or strongly enhance the exiting proton beam collimation, even overcoming the hot electron pressure and allowing for proton beam divergence lower than 10°.

This has important implications, both for the understanding of the physics of focusing plasma devices and for applications of these ion sources to HED physics. Circular proton beams could be used to drive or enhance hydrodynamic implosions, while highly collimated proton beams can be used for efficient eating of samples and precision irradiation of material and biological samples at a distance.

An extremely relevant corollary of this work, that could have major implications in extreme field science and laboratory astrophysics, is the generation of macroscopic magnetic fields with amplitude exceeding 10 kilo-Tesla, obtained by compression of relatively mild, 0.5 kilo-tesla magnetic fields by the highly energetic TNSA plasma inside the cone cavity.

By studying the dynamics of TNSA proton beams in focusing devices, we also performed the first successful proton radiography of > 10 kT magnetic fields. This can provide a method to generate and study in controlled conditions the physics of highly magnetized plasmas.

**Figure 1:**
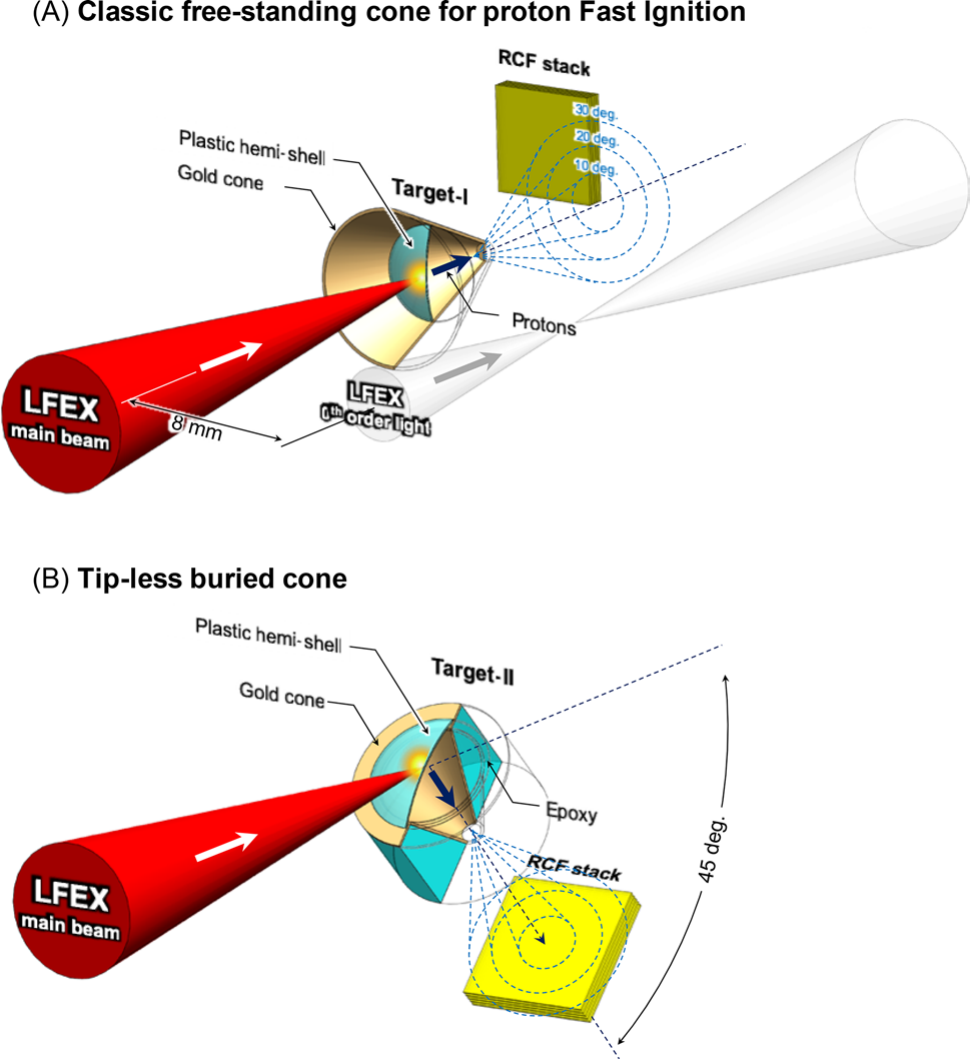
Schematic of the experimental setup. (**A**) Schematic of the classic free-standing cone as first proposed for proton fast-ignition. The LFEX laser is focused at normal incidence due to the cone geometry. The RCF-stack is positioned to the top-left quadrant in order to avoid the LFEX 0th order light (direct reflections from the compressor gratings). (**B**) Schematic of the tip-less buried cone target. The cone geometry allows for 45° laser incidence angle. In this way it is possible to avoid the risk of 0th order light irradiating the RCF stack and to collect the majority of the proton beam.

We investigate two types of cone targets as shown in (Fig. 1A,B), the first one is a classic ion fast ignition cone target, consisting of a free-standing gold cone with 50 µm tip and an hemi attached to the inner cone walls at 300 µm from the cone tip. The second one is a tip-less buried cone target, consisting of a tip-less gold cone 300 µm long embedded into a cylinder of Epoxy resin with diameter of 800 µm, and an hemi directly attached to the cylinder, coaxially with the cone at 300 µm from the cone tip.

The aperture at the cone tip is 50 µm in diameter and the radius of the hemi is 350 µm for both types of cones. The latter is rather similar to the cones investigated in the above-referenced works, but with smaller tip aperture. As main diagnostic we used radio-chromic film stack (RCF)^24,25^, as they can provide both spatially and energy resolved information on the generated proton beams.

### Experiment on LFEX laser

The experiment was performed on LFEX laser at the Institute of Laser Engineering, Osaka University. In this experiment, LFEX delivered 600 J of laser energy on target in 1.5 ps with four beamlets combined, for a nominal intensity of approximately 1 × 10^19^ W/cm^2^.

In case of classic free-standing cone, LFEX was focused at normal incidence on the hemi, along the cone axis direction. The RCF stack was aligned normally 25 mm from the cone tip and with the top-left corner adjacent to the cone axis.

This peculiar setup is required in order to avoid the RCF stack from being hit by the so-called “0^th^ order light”, constituted by the direct reflections of the uncompressed (2 ns) and partially compressed (10 ps) LFEX beams off the compressor gratings (due to the diamond compressor design), which are focused by the parabola about 8 mm to the side of the LFEX focus (see Fig. 1A).

The tip-less buried cone target geometry instead provides more flexibility and allows for large laser incidence angles as well. For this target we opted for 45 degrees incidence, which allows to collect the entire proton beam instead of a single quadrant as in the free-standing cone case, as schematized in Fig. 1B. Simple free-standing hemi target, identical to those attached to the cone targets and aligned at 45 degrees incidence from LFEX laser, were also investigated for comparison.

### Experimental results for free-standing cone targets.

**Figure 2:**
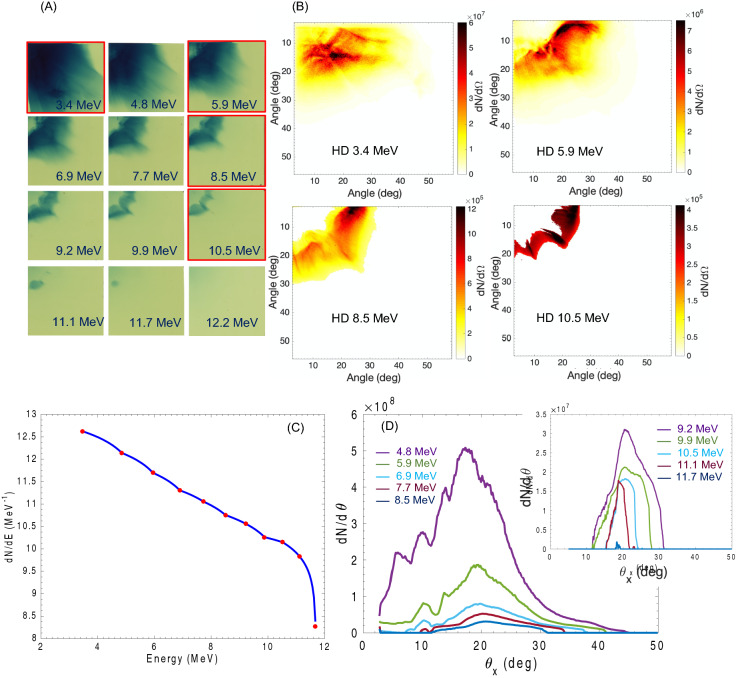
Example of experimental and post-processed data for free-standing cone target. (**A**) Raw RCF data showing the proton beam distribution for free-standing cone target. The RCF film corresponding to the post processed data in (B) are framed in red. (**B**) Post-processed data with 3-dimensional spectral unfolding showing the angularly resolved proton energy spectrum. The post-processed results are displayed in different color-scale to make them clear to the reader. The unit of solid angle $d\Omega$ is fixed at 7 × 10^–6^ sr. Clear ring-like pattern with large divergence is observed, with complete absence of protons in a 0.225 sr solid angle for energies > 6 MeV. At lower energy we can observe presence of protons emitted more at smaller angles. However, post-processed data show that even at low energies the distribution presents the ring-like pattern with average divergence angle > 10°. (**C**) Proton energy spectrum obtained from the spectral unfolding of the RCF data. (**D**) Angular distribution of the proton beam for all energies, the inlet image shows the angular distribution for protons with energy ≥ 9.2 MeV which are not clearly visible in the large format image.

Free-standing cone target-accelerated protons have a maximum energy of 12 MeV and display a clearly diverging annular pattern. The average divergence angle is comprised between 10 and 30°. For middle to high energies (E > 6 MeV), the quasi-totality of the protons is deflected at angles greater or equal than 10 degrees, while for lower energies we observe proton also at smaller angles. Original raw data, post processed data and analysis are displayed in Fig. 2. The apparent high density of protons at small angles in the lower energy films is due to the logarithmic response of the RCF (ionizing radiation-induced optical density). Post-processing of the image, however, reveals that the majority of the protons are emitted at angles greater than 10° even at low energy.

### Experimental results for tip-less buried cone targets.

**Figure 3:**
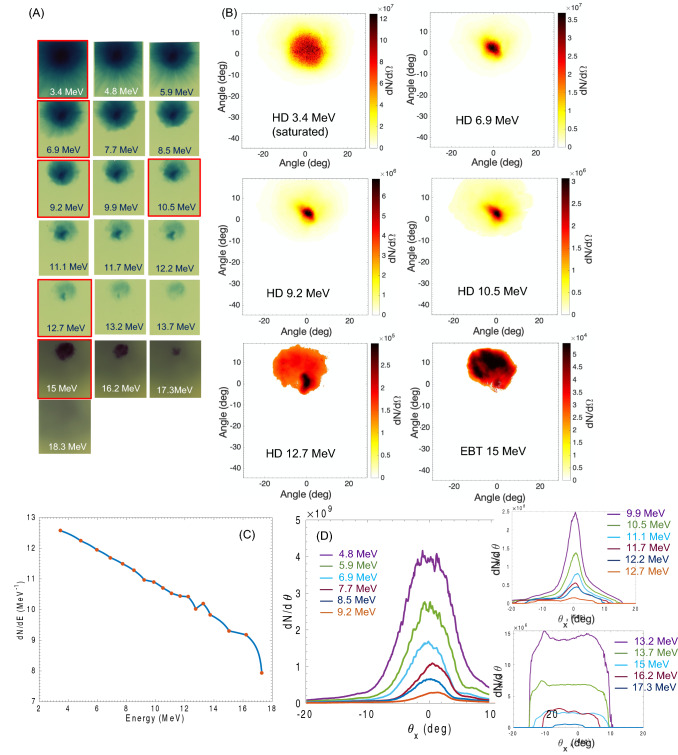
Example of experimental and post-processed data for tip-less buried cone target. (**A**) Raw RCF data showing the proton beam distribution tip-less buried cone target. The RCF film corresponding to the post processed data in (**B**) are framed in red. (**B**) Selection of post-processed data from the measurement in (**A**). It is evident the presence of a highly collimated proton beam component up to 13 MeV, characterized by divergence angles comprised between 11 degrees at low energy and 6.5 degrees at higher energy. Measurement on the first two RCF foils, corresponding to energies of 3.4 and 4.8 MeV is more difficult due to RCF saturation in the green channel at the beam center. For Energies exceeding 13 MeV the collimated component fades and gives place to a broader distribution with significantly higher divergence, starting at about 25 degrees, and reducing for higher energies similarly to typical TNSA proton beams. (**C**) Proton energy spectrum calculated from the spectral unfolding of the RCF data. (**D**) Proton beam angular distribution for all energies, clearly showing a divergence ≤ 11 degrees for proton energies between 4.8 and 9.8 MeV. The enclosed plots represent the angular distribution for (up) the mid-energy component, showing that the collimation is maintained till 12.7 MeV and the high high-energy component, with higher divergence (down) typical of classic TNSA proton beams.

Tip-less buried cone targets yielded very different results. From the RCF stack data we can observe that the proton beam presents a highly collimated component that extends from low energies to about 13.5 MeV, followed at higher energies by a component with larger divergence, the latter containing only about 0.1% of the proton beam energy (see Fig. 3A–C). The highly collimated component has divergence angles comprised between 11 degrees at low energy and 6 degrees at high energy as shown in Fig. 3D. These values are far lower than the typical ones for TNSA accelerated protons, where the lowest divergence, corresponding to the highest energy protons, for maximum proton energies of 15–20 MeV is about 10 degrees half-angle^10,26,27^, while the majority of the beam is distributed over much wider angles up to 25–30 degrees half-angle for the lower energy component.

From the RCF post-processing analysis we find that a total of 6.47 × 10^12^ protons have been accelerated for a total beam energy of 5.3 J (about 1% laser-to-proton energy conversion efficiency), resulting in an average current density of 2 × 10^9^ A/cm^2^, which can be efficiently used for material sample and plasma heating in HED physics experiments.

## Discussion

In the previous section we have shown that classic free-standing cone targets and buried cone targets yield very different results in terms of proton beam dynamics and exiting beam divergence. The first produces proton beams with a ring-like spatial profile and divergence comprised between 10 and 30 degrees, while the second produces highly collimated proton beams with divergence comprised between 6 and 11 degrees for the majority of the spectrum.

**Figure 4:**
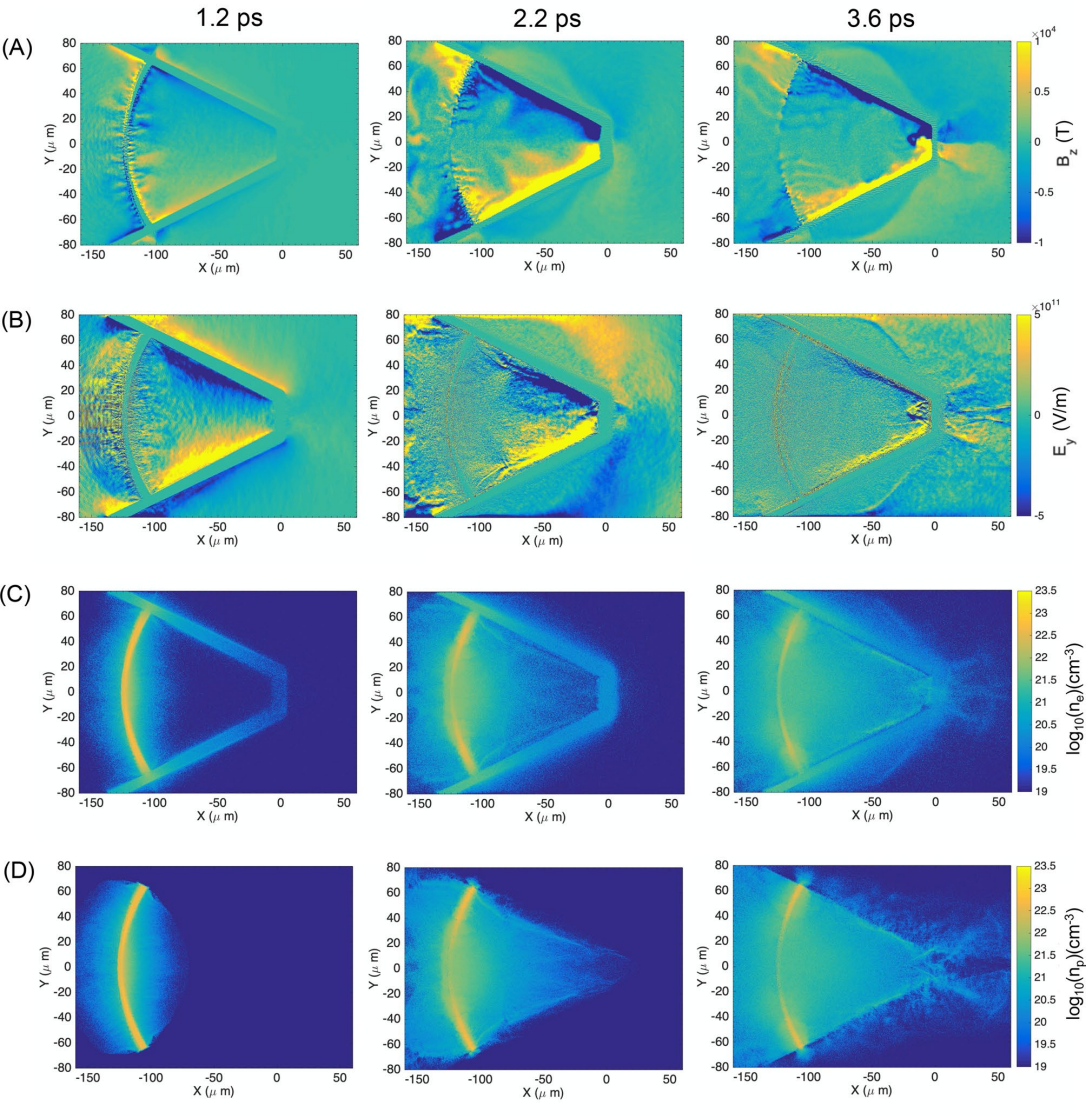
Bi-dimensional PIC simulations of proton beam generation and dynamics in free-standing cone target (**A**) Map of the Bz component of the magnetic field in the simulation for three simulation times: 1.2, 2.2 and 3.6 ps. At 1.2 ps, the surface current-generated magnetic field is initially distributed in the inner volume of the cone target. At later times it is compressed against the cone walls and tip by the expanding TNSA plasma, with magnetic field amplitude exceeding 10 kT and sharp gradients. (**B**) Map of the Ey component of the electric field for simulation times: 1.2, 2.2 and 3.6 ps. At early times the Ey component is produced mainly by fast electrons propagating along the cone walls. At intermediate times (not represented here) the electric field fades as the fast electron flow reduces. At 2.2 ps, when the TNSA plasma compresses the magnetic field inside the cone, a sudden strengthening of the electric field occurs due to the charge density gradient induced by the hot electron confinement by the magnetic field. Modulating the magnetic field gradients, the Ey component presents a de-collimating configuration that helps, together with the B-field, to deflect the proton beam exiting the cone. (**C**) Hot electron density map at 1.2, 2.2 and 3.6 ps simulation times. Only electrons coming from the hemi-shell are visualized in these maps. Initially the electron beam propagates and refluxes within the hemi-shell and the cone structure accelerating the proton beam via TNSA and generating focusing electric fields along the cone walls. At 2.2 ps as the TNSA plasma compresses the B-field, we can clearly observe the enhanced electron density in the area corresponding to the B-field gradients and the reduced electron density inside the B-field region. This is at the origin of the charge density gradient leading to the enhanced electric field in (**B**). This configuration is still clearly visible at 3.6 ps simulation time, with electrons accumulating at the edges of the magnetic field both on the cone walls and tip. (**D**) Hemi-shell proton density map at 1.2, 2.2 and 3.6 ps simulation time. At 1.2 ps we observe the protons being accelerated at the front and rear side of the hemi-shell. At later times, as the proton beam propagates inside the cone, we can clearly appreciate the focusing effect of the Ey component at 2.2 ps. At 3.6 ps, the proton beam is being deflected at the cone tip by the combined action of the Ey and Bz components, ultimately resulting in ring-like spatial distribution with large divergence angle, as observed in the experimental results.

**Figure 5:**
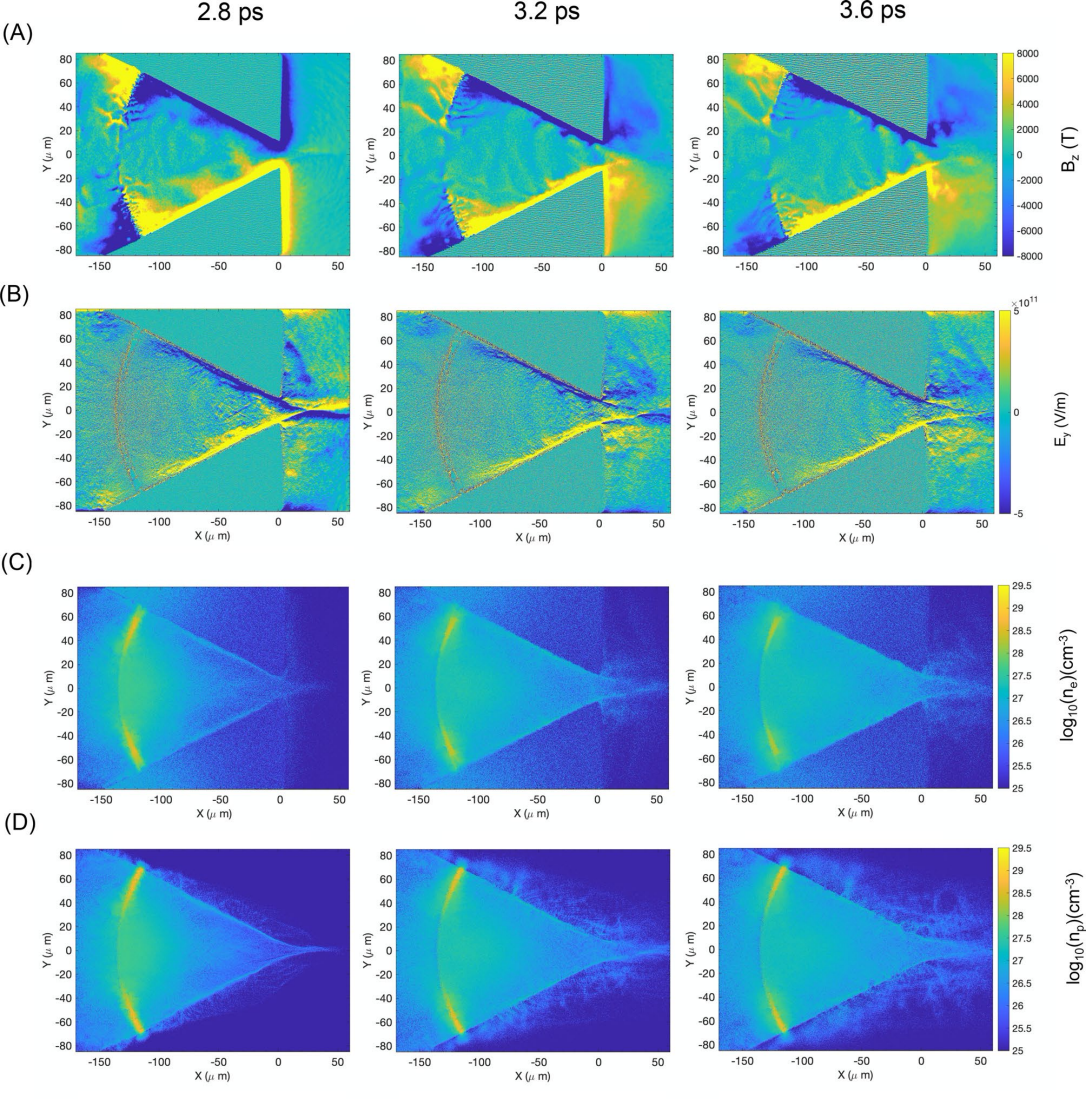
Bi-dimensional PIC simulations of proton beam generation and dynamics in tip-less buried cone targets. (**A**) Map of the Bz component of the magnetic field for simulation times of 2.8, 3.2 and 3.6 ps. At 2.8 ps we can observe the magnetic field compressed against the cone walls as in the free-standing cone case, with part of it flowing through the tip and along the outer rear surface of the target as the TNSA beam exits the cone. At 3.2 ps, as higher density TNSA plasma crosses the cone tip, part of the magnetic field is carried with the beam and forms a channel around it, which is maintained throughout the rest of the simulation. This B-field configuration prevents the hot electrons from radially expand and defocus the proton beam. (**B**) Map of the Ey component of the electric field for simulation times of 2.8, 3.2 and 3.6 ps. As the highest energy protons start exiting the tip at 2.8 ps, a strong de-focusing electric field surrounds the beam waist as result of the hot electron pressure. At later times of 3.2 and 3.6 ps we observe the disappearance of this de-focusing field, and the appearance of a focusing electric field structure in correspondence of the B-field gradient, preventing lateral expansion and allowing for a highly collimated beam to ensue. (**C**) Hot electron density map at 2.8, 3.2 and 3.6 ps. Despite the thick bulk material surrounding the cone, fast electrons are mostly confined in the cone cavity by the compressed magnetic field. At 2.8 ps we can see the hot electrons exiting the tip in a narrowly focused beam, following the proton density distribution. These electrons are responsible for the de-focusing electric field observed in (**B**). At later simulation times we observe the electrons being radially confined by the magnetic field structure, giving rise to the collimating electric field discussed in (**B**). (**D**) Proton beam density map at 2.8, 3.2 and 3.6 ps. At 2.8 ps we observe a tightly focused proton beam exiting the tip of the cone. These higher energy protons are going to be defocused by the hot electron pressure-generated electric field discussed above. At later simulation times, as the magnetic and electric field focusing structure develops, we observe that the majority of the proton beam is emitted with very low divergence, in full agreement with the experimental results.

In this section we discuss in detail the physics that leads to these two apparently antithetic results, and we will show that both behaviors descend directly from the complex interaction between the self-generated magnetic field inside the cone and the TNSA plasma, which is summarized in the bi-dimensional particle in cell simulation results in shown in Figs. 4 and 5.

Being this a very complex and dynamic process, we decided to add movies for the free-standing cone simulations as Supplementary material. The reader may want to view the movies entitled: “Supplementary_Bz_movie”, “Supplementary_Ex_movie”, “Supplementary_Ey_movie”, “Supplementary_Eden_movie” and “Supplementary_Pden_movie”, which refer respectively to the evolution of the z-component of the B-field B_z_, the x-component of the electric field E_x_, the y-component of the electric field E_y_, the electron density map n_e_ and the proton density map n_p_.

When a solid target is irradiated by a high intensity laser pulse, about half of the absorbed laser energy goes in the form of a bright relativistic electron beam that propagates inside the target and along its surface. In order to propagate, the relativistic electron current needs to be counterbalanced by a so-called return current composed by electrons from the target material.

When the target in question is a cone-attached hemi, a surface current of electrons will propagate from the cone walls to the hemi, replenishing the electron charge that left the hemi during laser-plasma interaction. This surface current manifest itself via the generation of macroscopic magnetic fields, both inside the target and along the target surface and filling the inner volume of the cone with a relatively low amplitude (0.5–1 kT) magnetic field (Fig. 4A, 1.2 ps). At the same time, the fast electron flow from the hemi to the cone walls produces charge-separation electric fields (Fig. 4B, 1.2 ps) that are considered the responsible for the proton beam enhanced focusing on cone targets, but as we will see in the following paragraphs, this only occurs in the initial stage of ion acceleration.

As the energetic TNSA plasma expands inside the cone, the magnetic field is compressed against the cone walls and the cone tip, resulting in sharp gradients with magnetic field amplitudes largely exceeding 10 kT (Fig. 4A, 2.2 ps). In these conditions, the hot electrons present in the TNSA plasma (although we talk about proton/ion beams, TNSA plasmas are in average charge neutral) cannot penetrate the B-field, as their average Larmor radius in > 10 kT magnetic fields is sub-µm (Fig. 4C, 2.2 ps).

This produces a charge density gradient, resulting in the enhancement of the electric field at the B-field/TNSA plasma interface, which is ultimately responsible for the proton beam focusing down the cone tip, given that the original charge separation electric field has been drastically reduced by the plasma filling the cavity.

By comparing the magnetic field (Bz), electric field (Ey), the electron density (n_e_) and proton density (n_p_) maps reported in the simulation results of Fig. 4A–D at simulation time of 2.2 ps, it appears clear that the TNSA plasma electrons are prevented from penetrating the magnetic field, which results in higher electron density in correspondence to the Bz gradient, and lower electron density in the region of high magnetic field. This electron density gradient enhances the Ey component of the electric field, which is maintained despite the plasma filling of the cone cavity. The proton beam reacts to the electric field and it is further guided towards the tip of the cone.

At later simulation time of 3.6 ps, the magnetic field is fully compressed at the cone walls and tip. The Ey component of the electric field follows the Bz gradient with two distinct effects on the proton beam. On one hand, the beam is directed and focused towards the cone tip. On the other hand, the electric and magnetic field configuration at the cone tip is de-collimating, and their combined action on the protons crossing the cone tip results in deflected proton trajectories in a ring-like shape with large divergence angle, as observed in the experimental data.

The electric field generated by the interaction of the TNSA plasma with the surface-current generated B-field not only influences the proton beam in terms of focusing and subsequent deflection at the cone tip, but it also causes their slow-down when exiting the tip. This is clearly visible (Fig. 1 in the Supplementary Material) by looking at the negative Ex component that increases when the TNSA plasma reaches the cone tip. This also explains the lower maximum proton energy observed for the free-standing cone target case compared to the tip-less buried cone case.

The physics for tip-less buried cone targets is in many ways like the free-standing cone one and it is determined by the interaction and interplay between the magnetic field and the TNSA plasma.

From the simulation results in Fig. 5, the extension and maximum amplitude of the B-field is lower compared to the free-standing cone, as part of it flows freely outside the tip and it is not confined in the cone. Also, for tip-less buried cone the fast electron current along the cone walls is reduced as a large fraction of them flows in the bulk plasma surrounding the cone. This also contributes to reduce the amplitude of the surface-current generated magnetic field.

Nevertheless, we clearly observe the stages of TNSA plasma expansion in the cone, the consequent B-field compression at the cone walls with the enhancement of the Ey component, providing efficient focusing up to the cone tip.

However, the dynamics radically changes at the cone tip region, where the absence of tip allows the protons to flow freely outside the cone, without B-field accumulation and compression at the tip. In order to better explain the physics at the cone tip, we display the simulation results in Fig. 5 at later times of 2.8, 3.2 and 3.6 picoseconds, where the relevant dynamics occurs.

At first, the highest energy tail of the proton beam exits the tip, maintaining the trajectory it had inside the cone, resulting in a narrowly focused beam (Fig. 5D at 2.8 ps).

However, the hot electron pressure gives rise to a de-focusing electric field (Fig. 5B–D at 2.8 ps), which leads to the proton beam expansion as it propagates further into vacuum.

This result agrees with the experimental data showing broader proton emission at very high energies, from 13.7 to 17.3 MeV, as in more classic TNSA data, and it is also in agreement with the above-referenced work by Bartal et al., where the proton trajectories exiting the cone are affected by the hot electron pressure, acting as a de-focusing agent that drives the proton beam lateral expansion as it propagates in vacuum.

At later times however, the dynamics significantly changes as the TNSA plasma carries part of the magnetic field outside the cone. As soon as the B-field follows the TNSA beam outside the cone tip, the de-focusing electric field at the proton beam waist disappears and is replaced by a focusing electric field structure, in correspondence to the magnetic field distribution outside the cone (Fig. 5A,B at 3.2 and 3.6 ps). The electric field is originated by the hot electron confinement provided by the magnetic field structure outside the cone (Fig. 5C at 3.2 and 3.6 ps).

This mechanism quenches the effect of the hot electron pressure and collimates a large fraction of the proton beam as it propagates into vacuum, giving rise to the high flux component observed in the experimental data.

**Figure 6:**
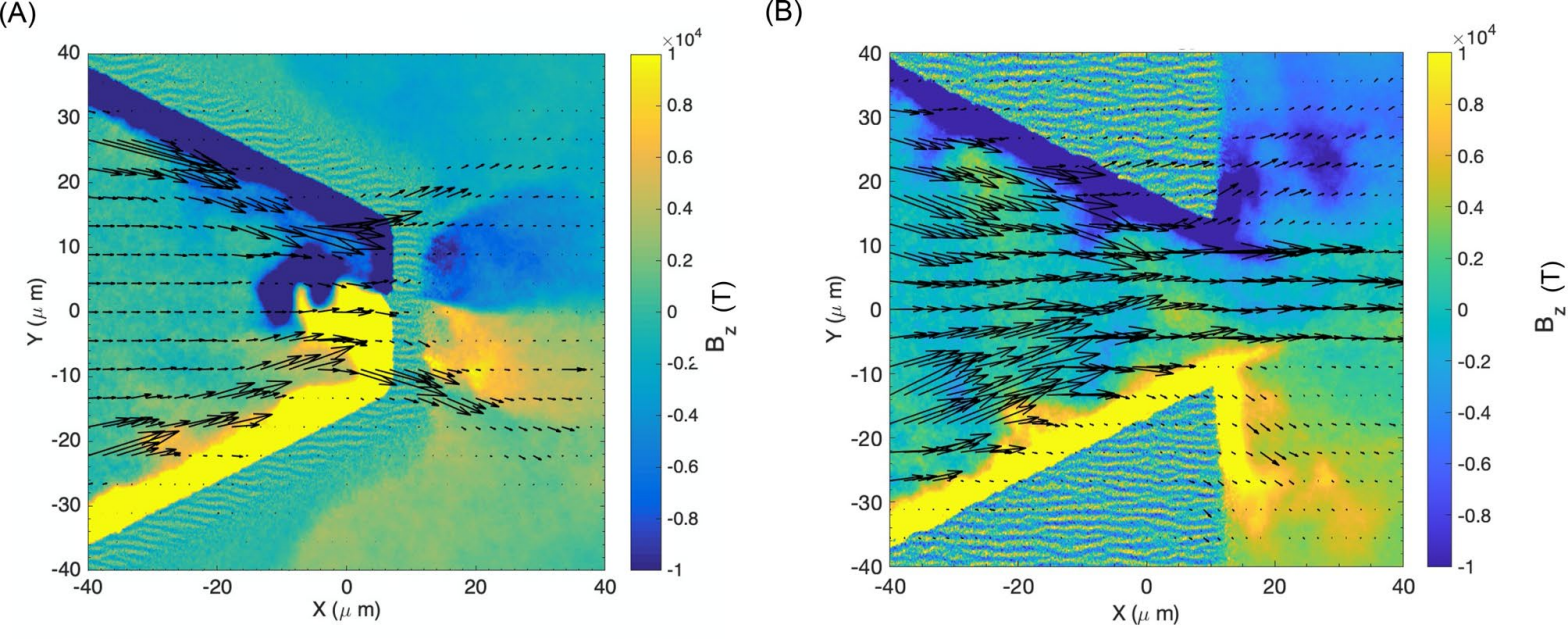
Map of the proton energy flux for free-standing and tip-less buried cone targets. (**A**) Proton energy flux superposed to the Bz map at 3.6 picoseconds in the simulation. It appears clear that the deflection of the proton beam occurs prior to reaching the tip due to the combined action of electric and magnetic field. (**B**) Proton energy flux superposed to the Bz map at 3.6 picoseconds in the simulation. It is evident that the majority of the protons exiting the cone tip are collimated by the azimuthal magnetic field structure and the consequent focusing electric field.

The proton beam dynamics at the cone tip region for the two types of targets is more easily understandable by looking at the proton energy flux maps shown in Fig. 6.

For free-standing cone target we can clearly observe the proton beam being guided to the cone tip, where is then split by the magnetic and electric field in two diverging directions (Fig. 6A), which in 3 dimensions would correspond to a ring-like emission. It is important to notice the splitting of the proton beam occurs inside the cone, in correspondence to the magnetic and electric fields, and it is not a post-emission effect related to the cone geometry itself.

For tip-less buried cone target (Fig. 6B), we observe the proton beam being focused at the tip and then further collimated outside the cone by the azimuthal magnetic field and the radial electric field distribution, preventing the beam from expanding due to radiation pressure, and resulting in a high-energy flux beam.

In summary, the mechanisms of proton beam focusing and emission from cone targets are determined by the self-generated magnetic field and its interaction with the expanding TNSA plasma.

For the focusing stage, the B-field helps maintaining a focusing electric field at the cone walls, even when the entire cone cavity is filled with plasma, by preventing the electrons from free-flowing to the cone walls and thus inducing hot electron density gradients that preserve and enhance the electric field. This is very different from the explanations provided in previous works, where the original TNSA-type electric field is considered the only responsible for the proton beam focusing.

For the emission stage, the magnetic field and the associated electric field determine the spatial profile and divergence of the proton beam by either accumulating at the cone tip and deflecting the incoming protons as in the free-standing cone target, or by flowing outside the cone, preventing the hot electron pressure from exerting radial pull on the proton beam and providing instead a collimating structure that allows the proton beam to propagate with minimum divergence.

In the work by Bartal et al., the cone is a tip-less buried type with very large aperture tip (127 µm), short distance between the hemispherical shell and the tip (150 µm) and larger aperture angle (60 degrees). This cone geometry allows for a large fraction of the proton beam to propagate unperturbed in the forward direction (especially the high-energy, low divergence protons), while only the higher divergence protons would be focused by the fields at the cone walls.

In addition, the experiment was performed on a laser of much smaller scale compared to LFEX (about 10% of the LFEX energy on target) with much reduced hot electron current density, lower amplitude self-generated magnetic field and much lower energy carried by the TNSA beam.

All these factors lead to a much-reduced energy density inside the cone, consequently the dynamics described in our publication does not manifest in Bartal and co-authors work.

On the other end, the effect of hot electron pressure-driven de-focusing of the proton beam, first described in Bartal’s work, is confirmed in our measurements and simulations as well (see Figs. 3D and 5B at 1.2 ps), with the highest energy part of the proton beam experiencing the radial pull exerted by the hot electron pressure, resulting in broader angular distribution at very high energies.

The results presented in our work have implications that go beyond the generation of high energy density states with ion and proton beams.

Control and collimation of laser-generated ion beams is being actively pursued by several research teams around the world. Tip-less buried cones represent a rather simple way to obtain collimated beam without requiring complex experimental setups. For smaller scale laser facilities, a key aspect would be to scale-down the cone target size while maintaining the aspect ratio, as to guarantee sufficient energy density inside the cone for the magnetic field-driven collimation to occur.

Moreover, it provides with a way to generate super-strong magnetic fields with amplitudes exceeding 10 kT, by taking advantage of the TNSA plasma pressure and treating it as a piston to compress the magnetic field, which is naturally generated as result of the fast electron return current. This can allow for relatively simple experimental setups with targets characterized by a partially enclosed volume, to allow for magnetic field compression, and some side windows/apertures to allow for diagnostics to peek-in and observe the physics of plasmas in super-strong magnetic fields, recreating conditions close those in the atmosphere of highly magnetized white dwarfs.

## Methods

### Experimental setup

The experiment was conducted on LFEX laser at the Institute of Laser Engineering, Osaka University. LFEX laser is composed by four beamlets and delivers up to 1 kJ of laser energy on target in 1.5 picoseconds, over a spot diameter of approximately 60 µm, resulting in an average intensity on target of 1 × 10^19^ W/cm^2^.

In this experiment the energy on target was limited to 600 J due to a limitation of the LFEX amplifiers output.

The cone targets are made of gold 10 µm thick, with an aperture angle of 45-degrees and tip size of 50 µm. For the tip-less buried cone, a thick Epoxy resin wall is added, giving this target the aspect of a cylinder with 800 µm base diameter and height of 300 µm.

The hemispherical shell, made of CH plastic has a radius of curvature of 350 µm and cross-sectional diameter of 300 µm. CH plastic was chosen because of the hydrogen-rich bulk material as LFEX laser is capable of fully depleting the contaminant layer of hydrocarbons that would constitute the proton source in metallic targets.

The diagnostic used was a RCF stack composed by 15 HD-V2 films followed by 20 EBT3 films. The stack was positioned at 2.5 cm distance from the target, and it was shielded with 105 µm Al foil which would protect the films from target debris. The LFEX incidence angle on target was either normal incidence for free-standing cone targets or 45-degrees incidence for the tip-less buried cone targets as shown in Fig. 1A and B.

### Data and statistical analysis

For data analysis and statistics, we refer to our recent publication in Review of Scientific Instruments^25^, describing the dosimetry calibration of Gafchromic HD-V2, MD-V3 and EBT3 films, that we briefly summarize in this section.

Dosimetry calibration was performed by irradiating the RCF films with a 130 Tera-Becquerel Co^60^ g-ray source with different exposure time, corresponding to radiation doses ranging from 1 Gy to 100 kGy.

The data were scanned using a response-calibrated Epson GT-X980 flatbed film scanner, that allows to calculate the optical density associated to the dose in each RCF and to obtain the optical density-to-dose calibration curves in red, green and blue channels.

For RCFs, the highest optical density is recorded in the red channel, however for high-dose exposures the red channel is not the best option given lower saturation threshold together with solarization effect that occurs for extremely high doses and that could lead to underestimation of the dose in the film. Therefore, the experimental data presented in this work are analyzed in the green channel, with an error associated to the calculated dose of 7.1% for HD-V2 and 5.1% for EBT3. To this error must be added the one associated to the batch-to-batch variation in RCF response as declared by Ashland-Gafchromic, corresponding to 20%.

Once the dose per RCF is obtained, data post-processing is performed via three-dimensional spectral unfolding procedure, entirely based on a method developed by Schollmeier and co-authors^28^. The post-processing code accounts for low-energy-transfer as well as straggling during transport in the RCF stack, providing as result the proton beam energy spectrum and angular distribution.

### Particle in cell simulations

Particle in Cell simulations have been performed with the Epoch2d code^29^ using two different simulation setups according to the different cone geometries and laser-plasma interaction conditions. The simulation box was 230 µm in the longitudinal dimension and 170 µm in the transverse dimension, with cell size l/30 in both dimensions. The cone walls have been modeled as Au18^+^ with density of 60 n_c_ and the hemi as pure hydrogen with density of 40 n_c_ and a sharp, 2 µm scale-length pre-formed plasma. A thin, 0.25 µm contaminant layer of hydrogen is also set on all the inner and outer cone surfaces.

The choice of pure hydrogen instead of CH plasma as hemi-shell material is since in relativistic laser-plasma interaction the laser energy absorption occurs through collisionless mechanisms, therefore no significant difference is expected between the two materials in terms or proton generation. Moreover, our experimental data are only related to protons, as the heavier ion stopping power is much higher compared to hydrogen and they are entirely stopped within the aluminium filter in front of the RCF pack.

## Supplementary Information

Supplementary Legends.

Supplementary Movie 1.

Supplementary Movie 2.

Supplementary Movie 3.

Supplementary Movie 4.

Supplementary Movie 5.

## Data Availability

All data needed to evaluate the conclusions in the paper are present in the paper and/or the Supplementary Materials. Data are stored at ILE and can be made available upon reasonable request.
