# Supplementary material for: Super-strong magnetic field-dominated ion beam dynamics in focusing plasma devices
Source: Sci Rep. 2022 Apr 27;12:6876. doi: 10.1038/s41598-022-10829-1 (PMC9046386; doi:10.1038/s41598-022-10829-1)
Supplement: Supplementary file 1 — Supplementary Legends. [file 41598_2022_10829_MOESM1_ESM.docx]

Supplementary video legends:

**Supplementary_Movie_M1**: Movie of 2D-PIC simulation results for free-standing cone target showing the evolution of the magnetic field component Bz from 200 fs to 3.6 ps.

**Supplementary_Movie_M2**: Movie of 2D-PIC simulation results for free-standing cone target showing the evolution of the Ey component of the electric field from 200 fs to 3.6 ps.

**Supplementary_Movie_M3**: Movie of 2D-PIC simulation results for free-standing cone target showing the evolution of the Ex component of the electric field from 200 fs to 3.6 ps.

**Supplementary_Movie_M4**: Movie of 2D-PIC simulation results for free-standing cone target showing the evolution of the hemi-shell electron density from 200 fs to 3.6 ps.

**Supplementary_Movie_M5**: Movie of 2D-PIC simulation results for free-standing cone target showing the evolution of the hemi-shell proton density from 200 fs to 3.6 ps.
